# Immune Subtypes in LUAD Identify Novel Tumor Microenvironment Profiles With Prognostic and Therapeutic Implications

**DOI:** 10.3389/fimmu.2022.877896

**Published:** 2022-06-03

**Authors:** Feng Wang, Xuan Gao, Peiyuan Wang, Hao He, Peng Chen, Zhentian Liu, Yujie Chen, Hang Zhou, Weijie Chen, Xin Yi, Xuefeng Xia, Shuoyan Liu

**Affiliations:** ^1^ Department of Thoracic oncology surgery, Fujian Medical University Cancer Hospital, Fujian Cancer Hospital, Fuzhou, China; ^2^ State Key Laboratory of Microbial Resources, Institute of Microbiology, Chinese Academy of Sciences, Beijing, China; ^3^ GenePlus-Shenzhen Clinical Laboratory, Shenzhen, China; ^4^ Fujian Key Laboratory of Translational Cancer Medicine, Fuzhou, China; ^5^ Department of Translational Medicine, GenePlus-Shenzhen Clinical Laboratory, Shenzhen, China; ^6^ Department of Translational Medicine, Geneplus-Beijing Institute, Beijing, China

**Keywords:** lung adenocarcinoma, prognostic signature, TME, immune subtypes (ISs), precision medicine

## Abstract

The six transcriptomic immune subtypes (ISs) (C1 - C6) were reported to have complex and different interplay between TME and cancer cells in TCGA (The Cancer Genome Atlas) pan-cancer cohort. Our study specifically explored how the consequence of interplay determines the prognosis and the response to therapy in LUAD cohorts. Clinical and molecular information of LUAD patients were from TCGA and Gene Expression Omnibus (GEO). The immune cell populations and gene/pathway enrichment analysis were performed to explore the molecular differences among the C3 IS and other ISs in the LUAD population. The proportion of C3 inflammatory IS was identified as the most common IS in both TCGA (*N =* 457) and GEO (*N =* 901) cohorts. The C3 IS was also found to be the most accurate prognostic subtype, which was associated with significantly longer OS (p <0.001) and DFS (p <0.001). The C3 IS presented higher levels of CD8 T, M1 macrophage, and myeloid dendritic cells, while lower levels of M2 macrophages and cancer-associated fibroblast cells. Moreover, the C3 subtype was enriched in the antigen process and presenting, interferon-gamma response, T cell receptor signaling, and natural killer cell-mediated cytotoxicity pathways than C1/C2. In contrast, the C1/C2 presented greater activation of pathways related to the cell cycles, DNA repair, and p53 signaling pathways. The immune-related C3 IS had a great ability to stratify the prognosis of LUAD, providing clues for further pathogenic research. This classification might help direct precision medicine screenings of LUAD patients, thus possibly improving their prognoses.

## Introduction

According to the cancer incidence report in GLOBOCAN 2018, lung cancer remains the leading cause of cancer incidence and mortality worldwide^1^. And lung adenocarcinoma (LUAD) is the most frequent histological subtype of non-small-cell lung carcinoma (NSCLC), accounting for 55% (1, 2). LUAD is a heterogeneous disease with variable clinical prognosis and drug response outcomes. However, the essential role of the immune system activating status in the development and progression of the tumor genome and heterogeneous has not been well characterized (3, 4).

Immune checkpoint inhibitors (ICIs) have been used to activate the anti-tumor T cell activation to obtain a durable cure for patients (5–7). But only 30% of LUAD patients could benefit from ICIs (6). LUAD is a heterogeneous disease with complex clinical features, biological diversity, and dynamic nature. Therefore the molecular classifications and therapeutic implications remain to be further studied (8). The expression level of PD-L1 protein on the tumor was reported as a predictive biomarker of poor prognosis of NSCLC (9, 10) and clinically benefiting from ICIs (11–14). PD-L1 is rich in benefits but is an imperfect marker, for the best response rate is still less than 50% in PD-L1 high NSCLC patients. Recently, anti-tumor immunity of LUAD patients (15–17), tumor immunogenicity (5, 6, 10–12, 16, 18–20), and tumor immune microenvironment (TME) (7, 11, 14, 18, 19, 21) are also reported to modulate the therapeutic impact of ICIs. Several biomarkers, including tumor mutational burden (TMB) (11), blood tumor mutational burden (bTMB) (22), human leukocyte antigens (HLA) loss-of-heterozygosity (LOH) status (20), and murine double minute 2/4 (MDM2/MDM4) amplification (23), which could affect the adaptive immune response to the tumors, have been widely studied to be associated with the efficacy of ICI therapy in LUAD patients.

Crosstalk between cancer cells and TME is sophisticated, comprising pro-tumorigenic and anti-tumorigenic manners. Therefore, it is still essential to further study the functional presentation of tumor neoantigen and the TME (3, 4) to predict the prognosis more precisely and improve the clinical outcome. Thorsson et al. classified the patients of 33 cancer types, including NSCLC, into six immune subtypes (ISs) (C1 - C6) (24). This study provided a resource for understanding tumor-immune interactions, with implications for identifying ways of advancing immunotherapy research. These six ISs were reported to be associated with overall survival (OS) and progression-free survival (PFS) (24). The C3 IS (inflammatory) heralded the best prognosis, while the C2 (IFN-g dominant) and C1 (wound healing) subgroups indicated less favorable outcomes despite having a substantial immune component. Moreover, the more mixed-signature subtypes, C4 (lymphocyte depleted) and C6 (TGF-b dominant), had the least favorable outcome. However, different cancer types had unique distributions of six ISs and prognosis. Thus, further refinement of this classification that was precisely adjusted for LUAD is undoubtedly warranted. Individual cancer types had varied proportions of ISs and clinical features, and the distinct lung cancer cells and tumor microenvironment of six ISs had complex and different interplay. The consequence of the complex interplay determines the growth of tumor cells and the prognosis of patients (24). In this context, the molecular characteristics describing tumor-immune effects remained unclear in LUAD. The investigation of molecular classifications at the multi-omics level could provide more insight into anti-tumor immunity and might acquire novel biomarkers.

**Figure 1:**
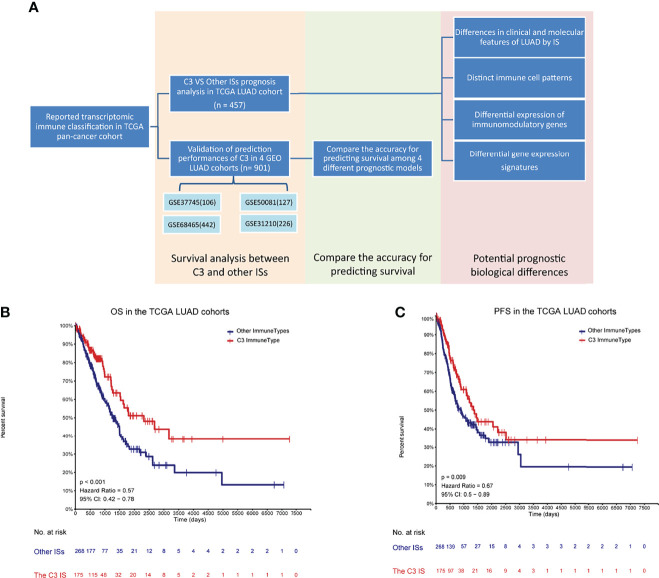
Developments and validations of the C3 immune subtype (IS) **(A)** Flowchart of developments and validations of the C3 immune subtype (IS). Overall survival (OS) **(B)** and progression-free interval event (PFI) **(C)** by immune subtypes (C3 vs. Other ISs) in TCGA LUAD cohort (n = 457) to verify the relationship between the C3 IS and prognosis. P-value was calculated by log-rank test.

In this study, we first identified the C3 IS as a robust prognostic signature associated with significantly longer overall survival and progression-free interval time by multivariate and subgroup analyses in multiple cohorts (TCGA LUAD cohort and four GEO LUAD cohorts). Then we further evaluated the prediction accuracy of the C3 IS and compared the C3 IS with the other three reported immune-related signatures. According to the time-dependent concordance index, we found that the C3 IS outperformed the other three signatures with superior overall survival and DFS predictive performances. Finally, using IS in this patient population, we analyzed the composition and functional orientation of immune and stromal populations of the tumor microenvironment, specific genes, and pathways. In conclusion, we aimed to provide more in-depth insight into the prognostic stratification of patients with LUAD and to provide a tumor-immune interaction profile with great promise of the therapeutic implications of LUAD (**Figure 1A**).

## Materials and Methods

### Study Population and Data Preprocessing

The mRNA expression counts data (Workflow Type: HTSeq-Counts) and clinical profiles of TCGA – NSCLC cohorts were downloaded from the PanCancer Atlas consortium, available at the publication page (https://gdc.cancer.gov/about-data/publications/pancanatlas) (25). Gene expression, copy number variation, and gene mutations were obtained for this study for 457 LUAD and 480 lung squamous cell carcinoma (LUSC) participants, grouped into five ISs based on the reported methods (24).

Four GEO datasets (901 LUAD participants) sourced from GEO databases with complete information about transcriptomics OS were also included in the validation dataset in our study (detailed in Supplementary Materials and Methods).

### Validate the Predictive Value of the C3 IS in GEO LUAD Cohorts

To further validate the predictive value of the C3 IS, Kaplan-Meier survival analysis and Cox proportional-hazard univariate and multivariate analyses were performed in four independent GEO LUAD data sets (GSE31210, GSE37745, GSE50081, and GSE68465) and, where five immune subtypes C1-C4, C6 were identified as the reported method.

### Comparison Between the C3 IS Prognostic Model and Other Three Reported Immune-Related Prognostic Signatures

To compare the prediction accuracies of C3 and the other reported prognostic models (**Table S2**), we used R package pec::cindex to calculate the concordance index (C-index) of all/each GEO independent LUAD cohort(s) (GSE31210, 114 GSE37745, GSE50081, and GSE68465) for detailed evaluations (26). OS and DFS time-dependent C-index were both calculated and compared.

### Clinical and Molecular Character Analyses

Associations of relevant known clinical and pathological prognostic factors and LUAD subtypes were assessed using Fisher’s exact test. Overall Survival (OS) was estimated according to the pairwise Kaplan-Meier method (27).

### Estimating Tumor Immune Score and Microenvironment Immune Cellular Fraction

The immune infiltration status of the tumor purity and immune components was computed using the Estimation of Stromal and Immune cells in MAlignant Tumours using Expression data (ESTIMATE) (28). The relative fraction of immune cells was estimated using CIBERSORT (http://cibersort.stanford.edu/) (29) and subtypes were obtained from the supplementary of a published paper (24). The cell estimated by the MCP-counter (30) were downloaded from TIMER2.0 (http://timer.cistrome.org/) (detailed in Supplementary Materials and Methods).

### Differentially Expressed Genes (DEGs) Analysis

We got the normalized expression levels of genes in FPKM values of the LUAD cohort. Processing of all the above data was done by the R (version 3. 6. 1). We used the R limma package to calculate the fold changes (FC) of the C3 subtype versus C1/2 subtypes. The Benjamini-Hochberg (BH) method was used for the adjusted p-value of multiple testing. A gene was defined as differentially expressed between IS subtypes when its median expression differed by at least 2-fold and multiple hypothesis correction of FDR p< 0.05.

### Gene Set Enrichment

Enrichment analysis was performed by cluster profile package. The version 7.1 Kyoto Encyclopedia of Genes and Genomes (KEGG) Genesets were obtained from the Molecular Signatures Database (MSigDB) (http://software.broadinstitute.org/gsea/downloads.jsp). The single-sample gene set enrichment analysis (ssGSEA) of signatures (median z-scores) in the three predominant immune subtypes of the TCGA LUAD (C3 versus C1/C2 dominant) were selected for each analysis, respectively, and used for heatmap visualization.

### Statistical Analysis

Kaplan-Meier survival analysis and Cox proportional-hazard univariate and multivariate analyses examined the significant difference between C3 and the other ISs in TCGA-LUAD and GEO cohorts. Associations of categorical variables and LUAD subtypes were assessed using Fisher’s exact test, and continuous variables and LUAD subtypes were compared through the Kruskal-Wallis or Wilcoxon signed-rank test.

## Results

### Prognostic Associations of Immune Subtypes in LUAD

To investigate the distribution of ISs in the TCGA NSCLC patients and determine their association with survival, we categorized 457 LUAD patients and 480 LUSC patients into five immune subtypes based on the reported methods (24). Only five ISs were identified in both the TCGA LUAD and LUSC cohort, with two predominant ones (the C2 IFN-γ dominant subtype [147 patients, 32.2%] and the C3 inflammatory IS [179 patients, 39.2%]) were present in TCGA LUAD patients. Moreover, the proportion of the C3 inflammatory IS (179 patients, 39.2%) was the most common IS observed in the TCGA LUAD cohort, whereas C1 was particularly dominant in LUSC (273 patients, 57.1%). Other ISs were less commonly encountered in the TCGA NSCLC cohort, such as the C1 Wound healing (83 patients, 18.2%), C4 Lymphocyte depleted (20 patients, 4.4%), and C6 TGF-β dominant (28 patients, 6.1%) subtypes in the LUAD cohort; C2 IFN-γ dominant (181 patients, 37.7%), C3 Inflammatory (four patients, 0.8%), C4 Lymphocyte depleted (seven patients, 1.5%), and C6 TGF-β dominant (14 patients, 2.9%) subtypes in the LUSC cohort (24). While the IS distribution in LUAD and LUSC cohorts was inconsistent, the C5 subtype was not identified in both cohorts (**Supplementary Table S1**).

In our immune study focused on NSCLC, the association of overall survival among ISs was still significant in the TCGA LUAD cohort (Log-rank *P* = 0.011) (**Figure S1A**), as Thorsson et al. reported in the pan-immune study, and the C3 performed the best prognosis. In contrast, the association was not significant in the TCGA LUSC cohort (Log-rank *P* = 0.14) (**Figure S1B**), and the C3 performed worse OS than the C1. This difference may course by the distinct distribution of C3, for there were only eight patients in the C3 subtype in TCGA LUSC cohort. Importantly, patients of the C3 IS had significantly longer OS and PFS in the TCGA LUAD cohort (Log-rank *P* < 0.001, HR = 0.57; *P =* 0.009, HR = 0.67, respectively) (**Figures 1B, C**). But the association of disease-free time (DFS) was not significant owing to the censored patients in the TCGA LUAD cohort (Log-rank *P =* 0.094, HR = 0.67) (**Figure S1C**). Univariate Cox regressions showed that pathological stage and the C3 IS were significantly associated with OS in the TCGA LUAD cohort (the Pathologic_stage: HR = 2.515, 95% CI 1.803-3.509, *P =* 0.001; the C3 IS: HR = 0.566, 95% CI 0.402-0.798, *P =* 0.001; respectively) (**Table 1**). Further, multivariate Cox regressions demonstrated that pathological stage and the C3 IS were independent prognostic factors (the Pathologic_stage: HR = 2.518, 95% CI 1.804-3.516, P < 0.001; the C3 IS: HR = 0.566, 95% CI 0.402-0.798, *P =* 0.001; respectively) (**Table 1**).

**Table 1:** Univariate and multivariate Cox analyses of risk factors for survival prediction in TCGA LUAD cohorts.

	Univariate analysis		Multivariate analysis	
	HR (95% CI)	P Value	HR (95% CI)	P Value
Age (>=median VS. <median)	1.161 (0.932-1.445)	0.183		
Gender (Female VS. Male )	0.921 (0.739-1.146)	0.459		
Pathologic_stage (III and IV VS. I and II)	2.515 (1.803-3.509)	0.001	2.518 (1.804-3.516)	< 0.001
Smoking status (Smoker VS Non-smoker)	0.801 (0.568-1.131)	0.207		
ImmuneType (C3 VS others)	0.566 (0.402-0.798)	0.001	0.566 (0.402-0.798)	0.001

### Independent Prognostic and Predictive Value of the C3 Immune Signature in Gout GEO LUAD Data Sets

**Figure 2:**
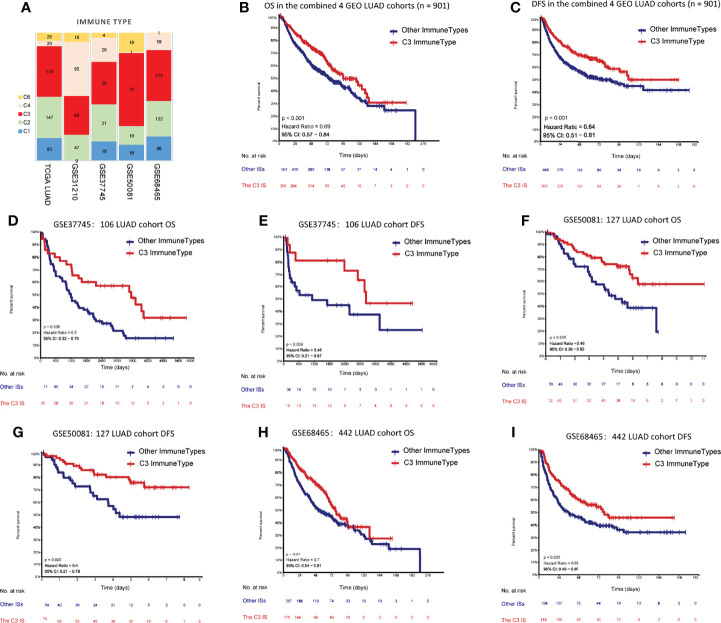
Prediction performances of the C3 IS in validation datasets. **(A)** Distribution of immune subtypes according to the immune subtypes in both TCGA and four GEO LUAD cohorts (*n=* 901). Kaplan-Meier analyses of OS between the C3 IS and the other ISs in the combined 4 GEO LUAD cohorts (*n =* 901) **(B)**, GSE37745 (*n =* 106) **(D)**, GSE50081 (*n =* 127) **(F)**, and GSE68465 (*n =* 442) **(H)**. Kaplan-Meier analyses of DFS between the C3 IS and the other ISs in the combined 4 GEO LUAD cohorts (*n =* 901) **(C)**, GSE37745 (*n =* 106) **(E)**, GSE50081 (*n =* 127) **(G)**, and GSE68465 (*n =* 442) **(I)**.

To examine whether the C3 IS was a robust molecular factor for survival prediction in the validation sets, univariate and multivariate Cox regressions were also carried out in four GEO LUAD cohorts (**Table 2**). In each of the four GEO validation data sets, patients were stratified into six ISs according to Thorsson et al.’s immune classification method. We used ImmuneSubtypeClassifier R-package (https://github.com/CRI-iAtlas/ImmuneSubtypeClassifier) which were pointed by the CRI iAtlas portal resources page (https://cri-iatlas.org/resources/) for classification of the LUAD immune subtype (24, 31). The proportion of C3 was also the most common IS observed in GEO LUAD cohorts (GSE37745, GSE50081, GSE68465), except for one stage I-II lung adenocarcinomas cohort (GSE31210) (**Figure 2A**; **Table S2**). Kaplan-Meier survival analyses showed that patients of the C3 IS had significantly longer overall survival time in the combined four GEO LUAD cohorts (n = 901), GSE37745 (n = 106), GSE50081 (n = 127), and GSE68465 (n = 442) cohorts (HR = 0.69, 95% CI 0.57-0.84, *P* < 0.001; HR = 0.5, 95% CI 0.32-0.79, *P* = 0.006; HR = 0.46, 95% CI 0.26-0.82, *P* = 0.005; HR = 0.7, 95% CI 0.54-0.91, *P* = 0.01; respectively) (**Figures 2B, D, F, H**). Patients of the C3 IS also had improved disease-free interval time in the combined four GEO LUAD cohorts (n = 901), GSE37745 (n = 106), GSE50081 (n = 127), and GSE68465 (n = 442) cohorts (HR = 0.64, 95% CI 0.51-0.81, *P* < 0.001; HR = 0.45, 95% CI 0.21-0.97, *P* = 0.056; HR = 0.40, 95% CI 0.21-0.78, *P* = 0.005; HR = 0.65, 95% CI 0.48-0.87, *P* = 0.005; respectively) (**Figures 2C, E, G, I**). But no significant overall survival or disease-free survival were observed between the C3 and the other ISs in the GSE31210 (n=226) cohort (**Supplementary Figure S2**).

**Table 2:** Univariate and multivariate Cox analyses of risk factors for survival prediction in 4 GEO LUAD cohorts.

	Univariate analysis		Multivariate analysis	
GEO OS validation set (n = 687)	HR (95% CI)	P value	HR (95% CI)	P value
age,years (>=median VS. <median)	1.537 (1.29-1.83)	0.001	1.548 (1.298-1.845)	< 0.001
gender (Male VS. Female)	1.163 (0.979-1.382)	0.085		
pathologic_stage (III and IV VS. I and II)	3.065 (2.426-3.873)	0.001	3.009 (2.378-3.806)	< 0.001
smoking_status (Smoker VS Non-smoker)	1.755 (1.389-2.217)	0.001	1.649 (1.303-2.086)	< 0.001
ImmuneType (C3 VS others)	0.782 (0.653-0.937)	0.008	0.761 (0.635-0.913)	0.003

Univariate Cox regressions also showed that younger age, lower pathological stage, non-smoking status, and the C3 IS were strongly associated with longer overall survival and disease-free interval time in the combined GEO LUAD validation set (the C3 IS: HR = 0.782, 95% CI 0.653-0.937, P < 0.008; HR = 0.719, 95% CI 0.599-0.863, *P =* 0.001; respectively) (**Tables 2**, **3**). After adjusting for clinical and pathologic factors, further multivariate Cox analysis suggested that the C3 IS was still a novel independent molecular indicator for predicting longer overall survival and disease-free interval time (HR = 0.761, 95% CI 0.635-0.913, *P =* 0.003; HR = 0.667, 95% CI 0.604-0.849, P < 0.001; respectively). This predictive value of the fact that the C3 IS led to a better outcome in LUAD, perhaps reflecting a balanced immune response of the C3 IS.

**Table 3:** Univariate and multivariate Cox analyses of risk factors for DFS prediction in 4 GEO LUAD cohorts.

	Univariate analysis		Multivariate analysis	
GEO DFS validation set (n = 632)	HR (95% CI)	P value	HR (95% CI)	P value
ImmuneType (C3 VS others)	0.719 (0.599-0.863)	0.001	0.667 (0.604-0.849)	<0.001
age,years (>=median VS. <median)	1.255 (1.056-1.492)	0.01	1.295 (2.012-3.268)	0.004
gender (Male VS. Female)	1.101 (0.927-1.307)	0.274		
pathologic_stage (III and IV VS. I and II)	2.902 (2.212-3.807)	0.001	3.141 (0.604-0.849)	<0.001
smoking_status (Smoker VS Non-smoker)	1.361 (1.111-1.668)	0.003	1.32 (2.012-3.268)	0.008

### Comparisons Between the C3 IS and Other Three Reported Immune-Related Prognostic Signatures in LUAD Cohorts

To further evaluate the prediction accuracy of the C3 IS, we compared the C3 IS with the other three reported immune-related signatures in three GEO LUAD cohorts (**Table S3**). Only GEO cohorts with sample sizes larger than 100 were used in model comparisons. Although the GSE31210 cohort had more than 100 patients, synchronously, it was an early-stage (stage I or II) cohort with driver mutations. Hence, we still excluded this cohort.

**Figure 3:**
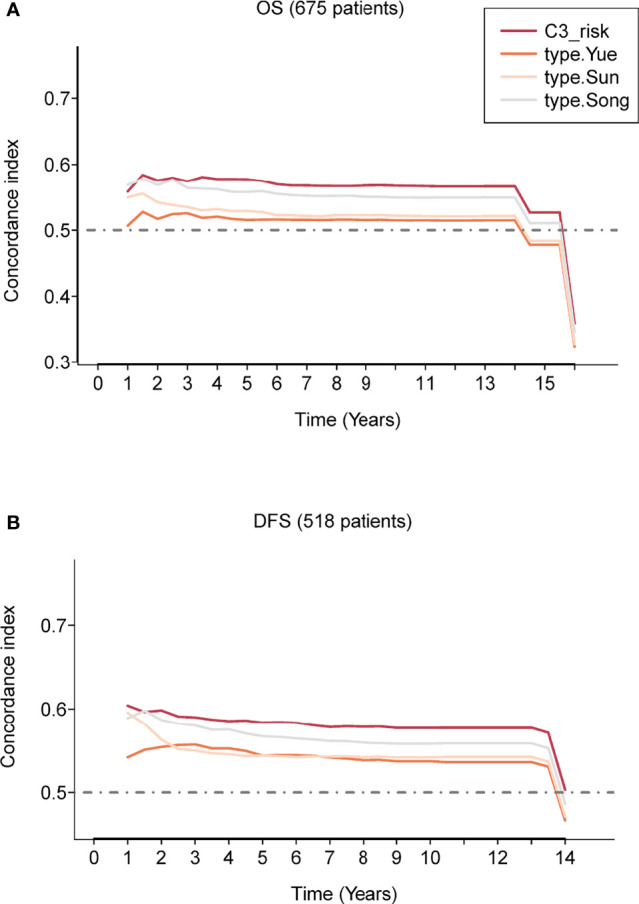
Prognostic performance evaluation of the C3 IS. Concordance index showing a measure of concordance of the predictor with OS **(A)** and DFS **(B)** between C3 IS and three reported prognostic models in the combined three GEO cohorts (*n =* 675).

First, we calculated the risk score for each patient in the three GEO cohorts by the three reported estimated regression coefficients retrieved from the respective studies using the expression data and divided patients into high-/low-risk groups with the median score. Then, we evaluated the concordance index for each survival time for the C3 IS and the other three reported models; the C3 IS outperformed the other three signatures with superior overall survival and DFS predictive performances according to the time-dependent Concordance index (**Figures 3**, **S3**). In all three prognosis prediction studies of LUAD, only immune-related genes from the ImmPort database were included (**Table S3**). The prognostic signatures were all only developed on lung adenocarcinoma patients. The limitations of these three reported signatures are obvious: they are only specific to lung cancers and cannot be applied to other cancer types.

### Clinical and Molecular Biomarkers of TCGA LUAD by ISs

**Figure 4:**
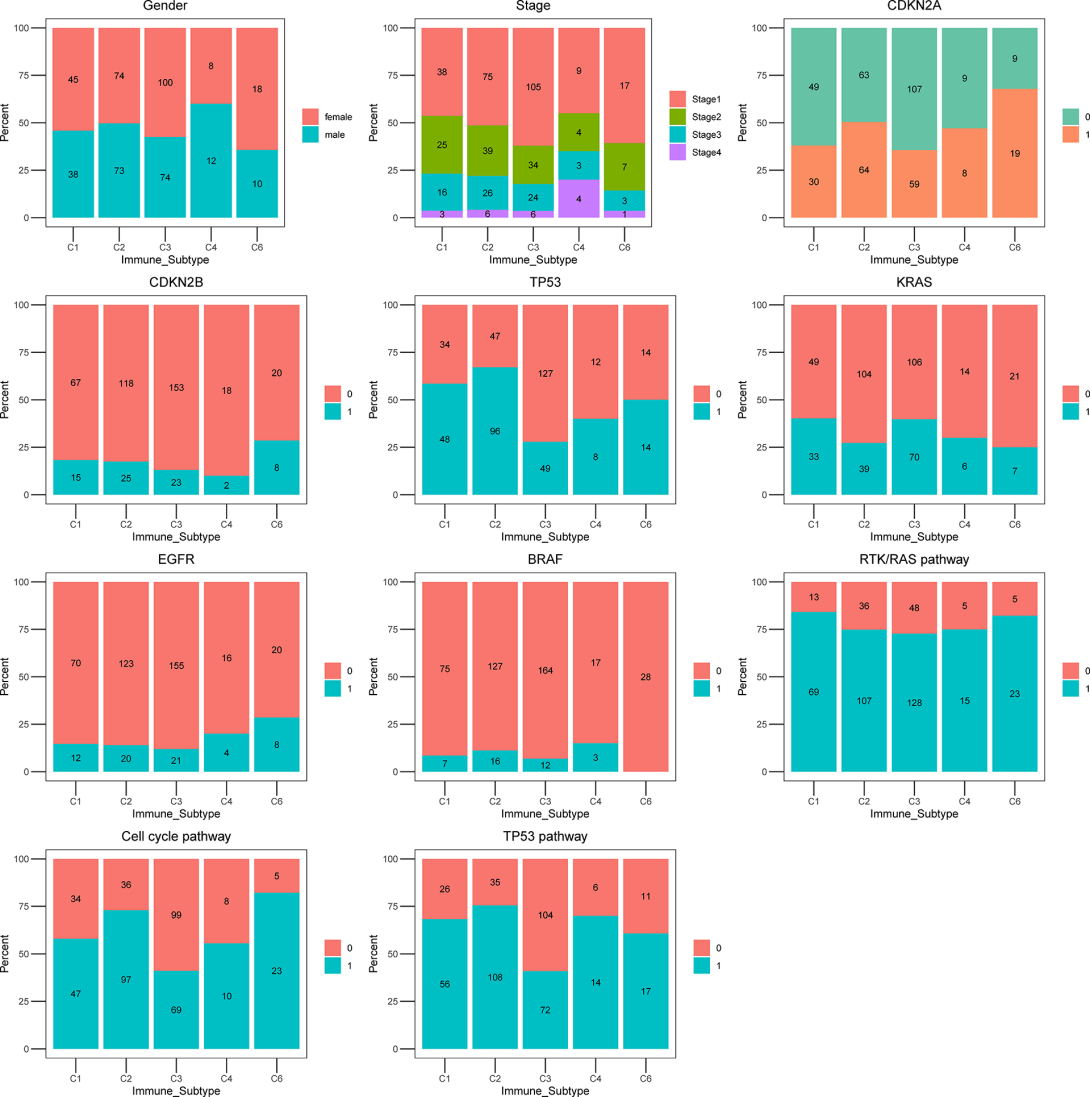
The proportion of clinical and molecular features of TCGA LUAD cohort according to five ISs. Bar plots showing the proportion of gender, stage, *CDKN2A*, CDKN2B, *TP53*, *KRAS*, *EGFR*, *BRAF*, RTK/RAS pathway, Cell cycle pathway, and *TP53* pathway in immune subtypes C1 (wound healing), C2 (IFN-g dominant), C3 (inflammatory), C4 (lymphocyte depleted) and C6 (TGF- β dominant).

To further characterize the clinical and molecular differences within ISs, the proportion of gender, clinical tumor stage, driver mutations, and critical pathways were included in the analysis (**Figure 4**). C3 IS was enriched in Stage I LUAD tumors, whereas C4 IS was frequently encountered in Stage IV. Overall survival was not significantly different among or/and between every two immune subtypes in the stage I TCGA LUAD cohort (**Supplementary Figures S4 A, B**). The distribution of stage I was also not significantly different within the immune subtypes in the TCGA LUAD cohort (Fisher’s exact *P* = 0.094; **Supplementary Figure S4C**). In summary, the clinical stage was not significantly associated with the C3 inflammatory IS (**Figure S4**).

All ISs presented a similar proportion of *KRAS* proto-oncogene, GTPase (*KRAS*) mutations, except for the C1 wound healing (39.76%) and C3 inflammatory (39.11%) ISs, where the highest fraction of *KRAS* mutations were identified. In the analysis of other reported prognosis-associated biomarkers, tumor protein p53 (*TP53*), B-Raf proto-oncogene, serine/threonine kinase (*BRAF*), cyclin-dependent kinase inhibitor 2A (*CDKN2A*), epidermal growth factor receptor (*EGFR*) mutations, cell cycle pathway, and TP53 pathway were most rarely observed in the C3 inflammatory IS (27.37%, 6.70%, 32.96%, 11.73%, 38.55%, 40.22%, respectively). Moreover, the *CDKN2A*, *TP53*, Cell cycle pathway, and TP53 pathway were mutated with significant differences among subgroups and were most rarely observed in the C3 inflammatory IS in the TCGA LUAD cohort (Fisher’s exact *P* = 0.005, *P* = 0.004, *P* < 0.0001, *P* = 0.0005, respectively) (**Figure 4**; **Supplementary Table S4**).

C3 IS is where the most common *KRAS* mutations were identified. In the analysis of other reported prognosis-associated biomarkers, tumor protein p53 (*TP53*), B-Raf proto-oncogene, serine/threonine kinase (*BRAF*), cyclin-dependent kinase inhibitor 2A (*CDKN2A*), epidermal growth factor receptor (*EGFR*) mutations, cell cycle pathway, and *TP53* pathway were most rarely observed in the C3 subtype. However, these figures are not particularly accurate in these last subgroups because of the small sample size.

### Estimating the Composition of Immune and Stromal Signatures Among C3 and Other Immune Subtypes

**Figure 5:**
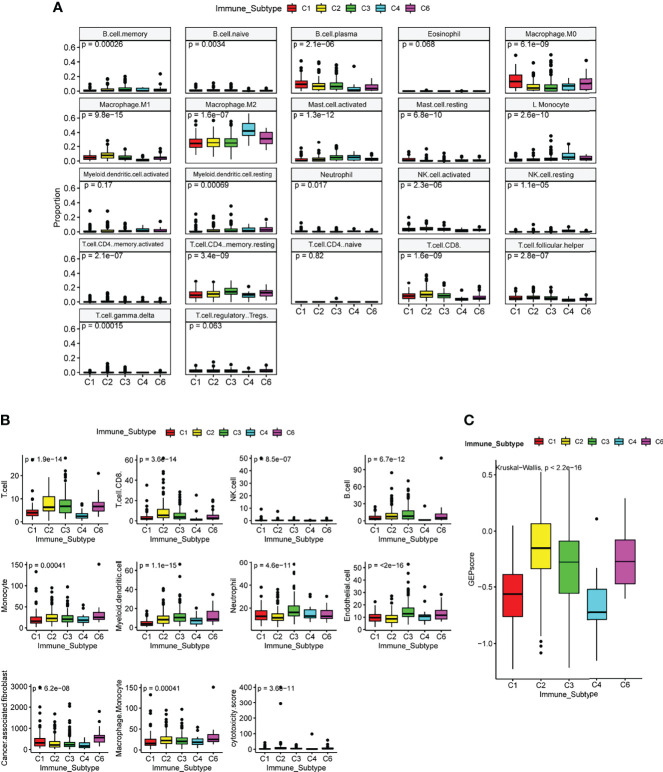
Immune and stromal cell populations of the immune subtypes in TCGA LUAD cohort. Immune and stromal signatures were estimated by CIBERSORT**(A)**, MCP-counter **(B)**, and GEP score **(C)** in LUAD patients by immune subtypes. P-value was calculated by the Kruskal Wallis test.

The tumor microenvironment and lung cancer cells of six ISs have complex and different interplay. The consequence of the interplay determines the growth of tumor cells and the prognosis of patients. To further evaluate the association of the C3 IS and immune infiltration in LUAD, we analyzed the immune and stromal signatures estimated using CIBERSORT and MCP-counter (29, 30). The C3 IS samples presented higher B cell memory, CD4 memory resting, and CD4 memory activating cells than the other ISs using CIBERSORT. The CD8 T, follicular helper T, and M1 macrophage cells showed a higher proportion in both C2 and C3 subtypes. Low levels of M2 macrophage cells were also found in the C3 subtype. No significant differences were found in eosinophil, myeloid dendritic activated, neutrophil CD4 T naïve, and Tregs cells within these five groups in ISs by using CIBERSORT (**Figure 5A**). According to the MCP-counter result, the C3 IS samples presented higher T, CD8 T, B, myeloid dendritic, neutrophils, and endothelial cells. Low levels of cancer-associated fibroblasts were also found in the C3 subtype (**Figure 5B**). GEP score were calculated and showed significantly different distribution in five ISs (Kruskal Wallis test, p < 0.0001) (**Figure 5C**). In summary, the C3 IS samples presented higher CD8 T and myeloid dendritic cells using both CIBERSORT and MCP-counter among the ISs. And the distribution trend of CD8 T, monocyte, and myeloid dendritic cells estimated by CIBERSORT and MCP-counter was similar among the ISs. Low levels of M2 macrophage cells were found in the C3 subtype. These findings indicated that the C3 IS is strongly linked with the adaptive immune response since it was closely related to both critical natural immunity-related components (dendritic, M1 macrophage, and neutrophil cells) and cytotoxic-related components (CD8 T cells).

### Immune Subtypes Show Differential Regulation of Immunomodulators and Pathway Signatures

**Figure 6:**
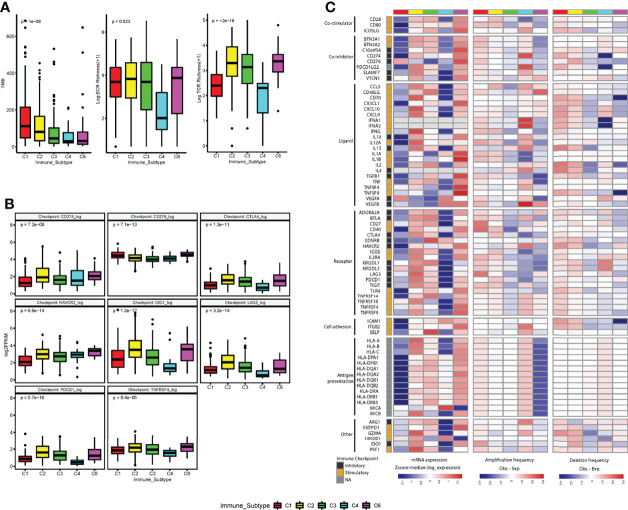
Regulation of immunomodulators. **(A)** The tumor immunogenicity (tumor mutational burden (TMB) and immune receptor repertoire (BCR and TCR diversity (log+1 transformed) are different across immune subtypes. **(B)** Distribution of expression levels for immune checkpoints (log2 FPKM) by immune subtypes. **(C)** Immune subtypes show differential expression of immunomodulatory genes. mRNA expression (log2FPKM), amplification frequency (difference between the observed versus the expected fraction of samples in which an IM is amplified), and deletion frequency (difference between the observed versus the expected fraction of samples in which an IM is deleted) for 75 immunomodulator genes by immune subtype. P-value was calculated by the Kruskal-Wallis test for immune checkpoints, TMB, BCR, and TCR analysis.

We first analyzed the differences between tumor immunogenicity and immune activation-related biomarkers (TMB, TCR richness, and BCR richness) among subtypes and found that TMB, antigen-specific T-cell receptor (TCR) richness, and B-cell receptor (BCR) richness, which determines the robustness of the anti-tumor response, were enriched in C2 compared with C3 IS (**Figure 6A**).

After that, we further analyzed the expression level of checkpoint genes and immunomodulatory genes to evaluate their role in shaping the TME across ISs. Although the gene expression levels of all the checkpoint genes showed significant differences among ISs, none of the immune checkpoint genes was significantly upregulated in the C3 IS (**Figures 6B**, **S5**).

To understand the state of expression and modes of control in different states of the TME across ISs, the gene expression and somatic copy number alterations (SCNAs, amplification, or deletion) of numerous immunomodulatory genes (IM) (24, 32) being evaluated in clinical oncology were analyzed. Gene expression of IMs (immunomodulatory genes) varied across ISs and played their role in shaping the TME. Upregulation of several stimulatory IMs was found after adjusted in C3 subtypes, such as CD27 molecule (*CD27*) (adjusted *p* = 0.0091), CD28 molecule (*CD28*) (adjusted *p* =0.0035), CD40 ligand (*CD40LG*) (adjusted *p* =2.5e^−14^), C-X-C motif chemokine ligand 10 (*CXCL10*) (adjusted *p* = 9.3e^−06^), interleukin 2 (*IL2*) (adjusted *p* = 0.0033), selectin P (*SELP*) (adjusted *p* = 3.10e^−16^), and TNF receptor superfamily member 14 (*TNFRSF14*) (adjusted *p* = 0.02). In contrast, downregulation of several inhibitory IMs was found in the C3 subtype, such as the CD276 molecule (*CD276*) (adjusted *p* =1.0e^−06^) (**Figure 6C**, **S5**).

**Figure 7:**
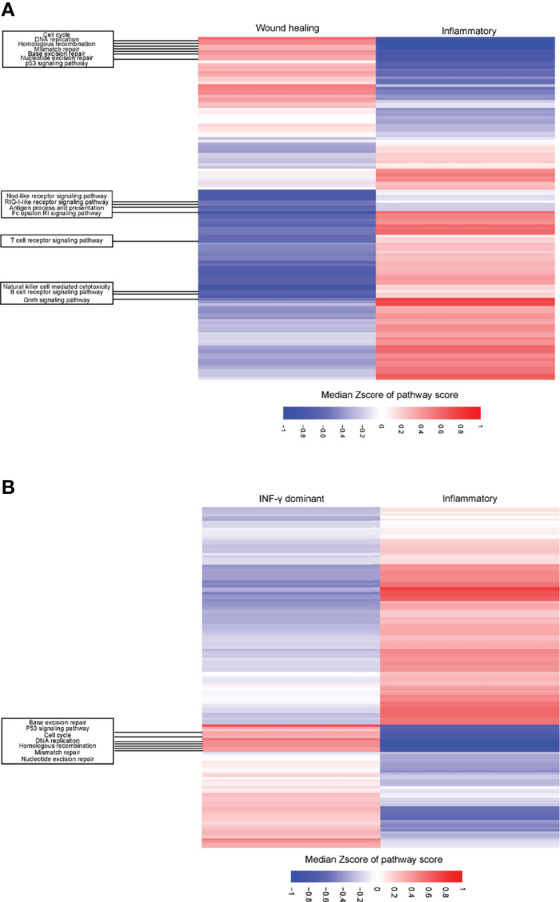
Differential expression of pathways between C1 (wound healing)/C2 (IFN-γ dominant) vs. C3 (inflammatory) immune subtypes in LUAD patients. **(A)** Heatmap showing the different upregulation of gene set enrichment analysis (ssGSEA) of KEGG pathways among the C1 (wound healing) and C3 (inflammatory) immune subtypes in the TCGA LUAD cohort. **(B)** Heatmap showing the different upregulation of gene set mRNA enrichment analysis (ssGSEA) of KEGG pathways among the C2 (IFN-γ dominant) and C3 (inflammatory) immune subtypes in the TCGA LUAD cohort. The result is expressed according to the median z-scores of the ssGSEA score of pathways.

GSEA showed that the C3 subtype had a heightened activation in antigen process and presenting pathway, interferon-gamma response pathway, T cell receptor signaling pathway, and natural killer cell-mediated cytotoxicity pathway compared to C1/C2 (**Figure 7**, **S6**). In contrast, the C1/C2 presented greater activation of pathways related to the cell cycles, mismatch repair (MMR), DNA repair, and p53 signaling pathways than the C3 subtype (**Figure 7**, **S6**).

## Discussion

Crosstalk between cancer cells and TME was sophisticated and still essential to be further studied (3, 4) to predict the prognosis more precisely and improve the clinical outcome of LUAD patients. Thorsson et al. developed a new pan-cancer immune classification in 33 solid tumor types and encompassed nearly all human malignancies and consists of six ISs with distinct immunogenomic features and clinical outcomes (24). This study provided a resource for understanding tumor-immune interactions, with investigations of molecular classifications at the multi-omics level, which could provide more insight into anti-tumor immunity and might offer novel biomarkers. Our study specifically characterized the C3 IS in LUAD patients and found that the C3 IS is a robust prognostic signature associated with significantly longer overall survival and progression-free interval time by multivariate and subgroup analyses in multiple cohorts (TCGA LUAD cohort and four GEO LUAD cohorts). And the evaluated prediction accuracies of the C3 IS outperformed the other three signatures with superior overall survival and DFS predictive performances. These multiple omics studies also investigated the underlying mechanism of how the consequence of interplay determines the prognosis and the response to therapy in LUAD cohorts. These results not only provided more in-depth insight into the prognostic stratification of patients but also the tumor-immune interaction of ISs with great promise for the therapeutic implications of LUAD.

The C3 inflammatory IS was particularly dominant in LUAD (179 patients, 39.2%) but far less common in LUSC (eight patients, 1.7%). We further characterized the association of the C3 IS and the clinical outcome in the TCGA cohort of LUAD patients, for the C3 IS was dominant in LUAD. Only five ISs were identified in both the TCGA LUAD and LUSC cohort, but the distributions of ISs in LUAD and LUSC were quite different. Thorsson et al. (24) reported two predominant ISs (the C2 IFN-γ dominant subtype [147 patients, 32.2%] and the C3 inflammatory IS [179 patients, 39.2%]) in TCGA LUAD patients, while the C1 wound healing subtype [275 patients, 56.6%] and C2 IFN-γ dominant subtype [182 patients, 37.5%] in TCGA LUSC patients. We also reported another relevant finding, the prognostic impact of the C3 IS in LUAD was consistent with the trend reported for the TCGA pan-cancer study. Consequently, we specifically studied the immune classification in LUAD patients and observed that the C3 IS was a robust immune-related phenotype with great prognostic performances in both TCGA LUAD patients and multiple LUAD cohorts in the GEO dataset. The C3 phenotype outperformed the other three reported signatures with superior overall survival and DFS predictive performances. To explore the mechanism of the significant influence of the C3 immune phenotype in survival, we further compared the heterogeneity of immune activity within five ISs. These findings may have relevant implications in terms of prognosis stratification and prediction of response to therapy and suggest the immune phenotype may allow a more accurate classification of patients to assist clinicians in personalized treatment.

In the past prognosis prediction studies of LUAD (33, 34), only immune-related genes were included. Sun et al. reported a four immune-related gene model immune-related prognostic signature for lung adenocarcinomas (IPSLUAD), an independent prognostic factor (34). Moreover, Guo et al. contributed a 10 immune-related genes signature for survival prediction and immune checkpoint molecules in lung adenocarcinoma. In contrast, we used a different selection method based on multi-omics level data reported in a TCGA pan-cancer analysis (24). And the C3 IS group was significantly associated with better DFS and OS by log-rank test, univariate, and multivariate Cox regressions in both TCGA and GEO LUAD cohorts. This trend was consistent with survival rates by ISs reported for the pan-cancer population. In each of the four GEO validation data sets, patients were stratified into six ISs according to Thorsson et al.’s immune classification method. The contribution of five ISs observed in GEO LUAD cohorts was consistent with the TCGA LUAD cohort, and the proportion of the C3 IS was also the most common one. Furthermore, the C3 IS outperformed the other immune-related signatures in both early-stage LUAD and multicenter cohorts. Therefore, the C3 IS could significantly supplement traditional staging systems and be an accurate clinical outcome predictor for patients with LUAD.

The distinct oncogene alterations could be related to different molecular classifications of LUAD. The most extensively reported and studied biological consequences and clinical implications (therapeutic, diagnostic, and prognostic) in LUAD were *EGFR*/*KRAS* alterations and *ALK* rearrangements in LUAD (35–37). And different driver mutations would significantly affect the response of target therapy, the ISs, and TME, making it essential to assess their interplay with the immune classification in LUAD. In addition, immune checkpoint inhibitors (ICIs) are widely used in the first-line treatment for NSLCL (38), and the system explores the distribution of ISs of relevant genomic and transcriptomic. The clinical outcome is needed. Cullis et al. reported that the oncogenic mutation of *KRAS* in NSCLC samples could increase the infiltration of CD8+ T cells and gain a better clinical outcome by ICIs treatment (37, 39). And the C3 subgroup was identified with the most common *KRAS* mutations among the ISs. In analyzing other reported prognoses and immune-associated biomarkers, the mutation rate of *TP53*, *BRAF*, *CDKN2A*, *EGFR*, cell cycle pathway, and TP53 pathway was most rarely observed in the C3 subtype. In conclusion, the C3 IS has potential for a better immune infiltration and TME, leading to a better prognosis and benefit from immunotherapy.

The immune landscape of LUAD showed that the C3 IS patients presented an upregulated immune activation and potentially impacted both the prognosis and the response to therapy. Given the fact that the C3 IS showed a higher distribution of several critical innate immunity-related components (dendritic, M1 macrophage, and neutrophil cells), tumor-specific cytotoxic killing components (CD8 T, follicular helper T, and myeloid dendritic cells estimated by both CIBERSORT and MCP-counter), and upregulation of several immune-stimulatory genes (*BTN3A1*, *CD27*, *CD28*, *CD40LG*, *CXCL1*, *CXCL1*0, *HMGB1*, *ICOSLG*, *IL2*, *SELP*, *TLR4*, and *TNFRSF14*). In addition, low levels of cancer-associated fibroblasts and M2 macrophages were found in the C3 subtype. This result is reasonable to explain that the C3 subgroup had better progress and may likely benefit from immune checkpoint inhibitors. These findings suggest that the better prognosis of the C3 IS may be contributed by activated innate immunity rather than tumor-specific cytotoxic killing. Despite that the prognostic impact of the C3 IS in LUAD was consistent with the reported result for the TCGA pan-cancer cohort, this excellent prognosis of inflammatory tumors is still unexpected and may help the precision therapy selection for patients.

Recently, TMB has emerged as an alternative biomarker. Studies have demonstrated its utility, irrespective of the PD-L1 level of a tumor (40). TMB is also an available biomarker in standard clinical practice to identify immunogenic ICB treatment in NCCN protocol in lung cancer. In our study, the C3 subtype contained the TMB higher than the C4 and C5 subtypes but lower than the C1 and C2 ISs. Meanwhile, the TCR diversity measured by species richness (41, 42) was higher than C1 and C4 ISs, but lower than the C2 and C6 ISs. These findings suggest that the C3 tumors showed low immunogenicity; in this situation, although T cells are present, low TMB and low neo antigenicity still impede their activity.

There still exist several limitations in this study. Our observations may be partially conditioned by some caveats inherent to the use of TCGA and GEO data. First is the fact that our analyses were limited by restriction to data from public databases, in the absence of treatments, follow-up information, and targeted classical cellular immunology assays for confirming cell phenotype distribution. Second, although the C3 IS was observed as a robust prognostic signature and presented an upregulated immune activation in the LUAD cohort, indicating these patients could potentially benefit from immunotherapy, future studies that would examine this hypothesis directly are still needed to confirm the findings of our bioinformatic analyses.

Importantly, our study first demonstrates that the C3 IS, a molecular classification at the multi-omics level, is a robust and prognostic stratificational signature by multivariate and subgroup analyses in multiple cohorts (TCGA LUAD cohort and four GEO LUAD cohorts). Moreover, the different functional orientations of immune and stromal populations in TME, and specific genes together with pathways among the ISs provide more in-depth insight into the prognostic stratification of LUAD patients and with great promise for therapeutic implications. We believe that these reported findings are highly relevant and should be considered for further exploration in the studies of future therapeutic strategies and may eventually improve the fate of LUAD patients.

## Data Availability Statement

The datasets presented in this study can be found in online repositories. The names of the repository/repositories and accession number(s) can be found in the article/**Supplementary Material**.

## Author Contributions

SL, XG, FW, and XX conceived and designed the study. XG, PW, and HH collected the data. FW, XG, PW, and PC performed data analysis. XG and FW wrote the paper. XX, XY, YC, HZ, ZL, and WC reviewed and edited the manuscript. All authors contributed to the article and approved the submitted version.

## Funding

This study was supported by the National Natural Science Foundation of China (Grant Number 82002497) and the Science and Technology Program of Fujian Province (Grant Number 2020J05072).

## Conflict of Interest

XG, ZL, XX, and XY are employees of Beijing GenePlus Technology Co., Ltd.

The remaining authors declare that the research was conducted in the absence of any commercial or financial relationships that could be construed as a potential conflict of interest.

## Publisher’s Note

All claims expressed in this article are solely those of the authors and do not necessarily represent those of their affiliated organizations, or those of the publisher, the editors and the reviewers. Any product that may be evaluated in this article, or claim that may be made by its manufacturer, is not guaranteed or endorsed by the publisher.

## Supplementary Material

Supplementary Figure 1 Prediction performances by immune subtypes in TCGA LUAD cohort. **(A)** Overall survival (OS) by immune subtype in the TCGA LUAD cohort (n = 457). P-value was calculated among subgroup stratification by log-rank test. **(B)** Overall survival (OS) by immune subtype in the TCGA LUSC cohort (n = 480). P-value was calculated among subgroup stratification by log-rank test. **(C)** Disease-free survival (DFS) by immune subtypes (C3 vs. Other ISs) in TCGA LUAD cohort (n = 260) to verify the relationship between the C3 IS and prognosis. P-value was calculated by log-rank test.

Supplementary Figure 2 Prediction performances by immune subtypes in the GEO cohort (GSE31210, n = 226). Overall survival (OS) **(A)** and Disease-free survival (DFS) **(B)** by immune subtypes (C3 vs. Other ISs) in the GEO cohort (GSE31210, n = 226) to verify the relationship between the C3 IS and prognosis. P-value was calculated by log-rank test.

Supplementary Figure 3 Prediction performances of the C3 IS and three reported models. Concordance index showing measure of concordance of predictor with OS and DFS between C3 IS and three reported prognostic models in the combined four GEO cohorts (n = 901) **(A–B)**, GSE37745 (n = 106) **(C)**, GSE50081 (n = 127) **(D)**, GSE68465 (n = 442) (E), and GSE31210 (n = 226) **(F).**

Supplementary Figure 4 The prognoses of stage I TCGA LUAD patients were not significantly different by the immune subtypes. **(A)** Overall survival by immune subtypes in the stage I TCGA LUAD cohort. Log-rank P-value was calculated among subgroup stratification. **(B)** Overall survival by immune subtypes in the stage I TCGA LUAD cohort. Log-rank P-value was calculated between every two subgroups. **(C)** Distribution of stages in the TCGA LUAD cohort by immune subtype. **(D)** Sankey diagram of stage and immune subtypes in the TCGA LUAD cohort.

Supplementary Figure 5 Differential expression of immunomodulatory genes between C3 IS and other ISs. The mRNA expression (log2FPKM) for 75 immunomodulator genes between the C3 (inflammatory) and other ISs in the TCGA LUAD cohort. Adjusted P-value was calculated for immune checkpoints.

Supplementary Figure 6 Differential expression of pathways between C1 (wound healing)/C2 (IFN-γ dominant) vs. C3 (inflammatory) immune subtypes in LUAD patients. Heatmap showing the details of different upregulation of ssGSEA of HALLMARK **(A)** and KEGG **(B)** pathways among the C1 (wound healing) and C3 (inflammatory) immune subtypes in the TCGA LUAD cohort. Heatmap showing the details of different upregulation of ssGSEA of HALLMARK **(C)** and KEGG **(D)** among the C2 (IFN-γ dominant) and C3 (inflammatory) immune subtypes in the TCGA LUAD cohort. The result is expressed according to the median z-scores of the ssGSEA score of pathways.

Supplementary Figure 7 Immune and stromal cell populations between the other ISs (A)/C2 (IFN-γ dominant) (B) and C3 (inflammatory) immune subtypes in the TCGA LUAD cohort. Immune and stromal signatures were estimated by CIBERSORT. And the P-value was calculated by the Wilcox test.
