# Supplementary material for: Immune Subtypes in LUAD Identify Novel Tumor Microenvironment Profiles With Prognostic and Therapeutic Implications
Source: Front Immunol. 2022 Jun 3;13:877896. doi: 10.3389/fimmu.2022.877896 (PMC9203850; doi:10.3389/fimmu.2022.877896)
Supplement: Supplementary Figure 1 — Prediction performances by immune subtypes in TCGA LUAD cohort. (A) Overall survival (OS) by immune subtype in the TCGA LUAD cohort (n = 457). P-value was calculated among subgroup stratification by log-rank test. (B) Overall survival (OS) by immune subtype in the TCGA LUSC cohort (n = 480). P-value was calculated among subgroup stratification by log-rank test. (C) Disease-free survival (DFS) by immune subtypes (C3 vs. Other ISs) in TCGA LUAD cohort (n = 260) to verify the relationship between the C3 IS and prognosis. P-value was calculated by log-rank test. [file DataSheet_1.pdf]

Figure S1

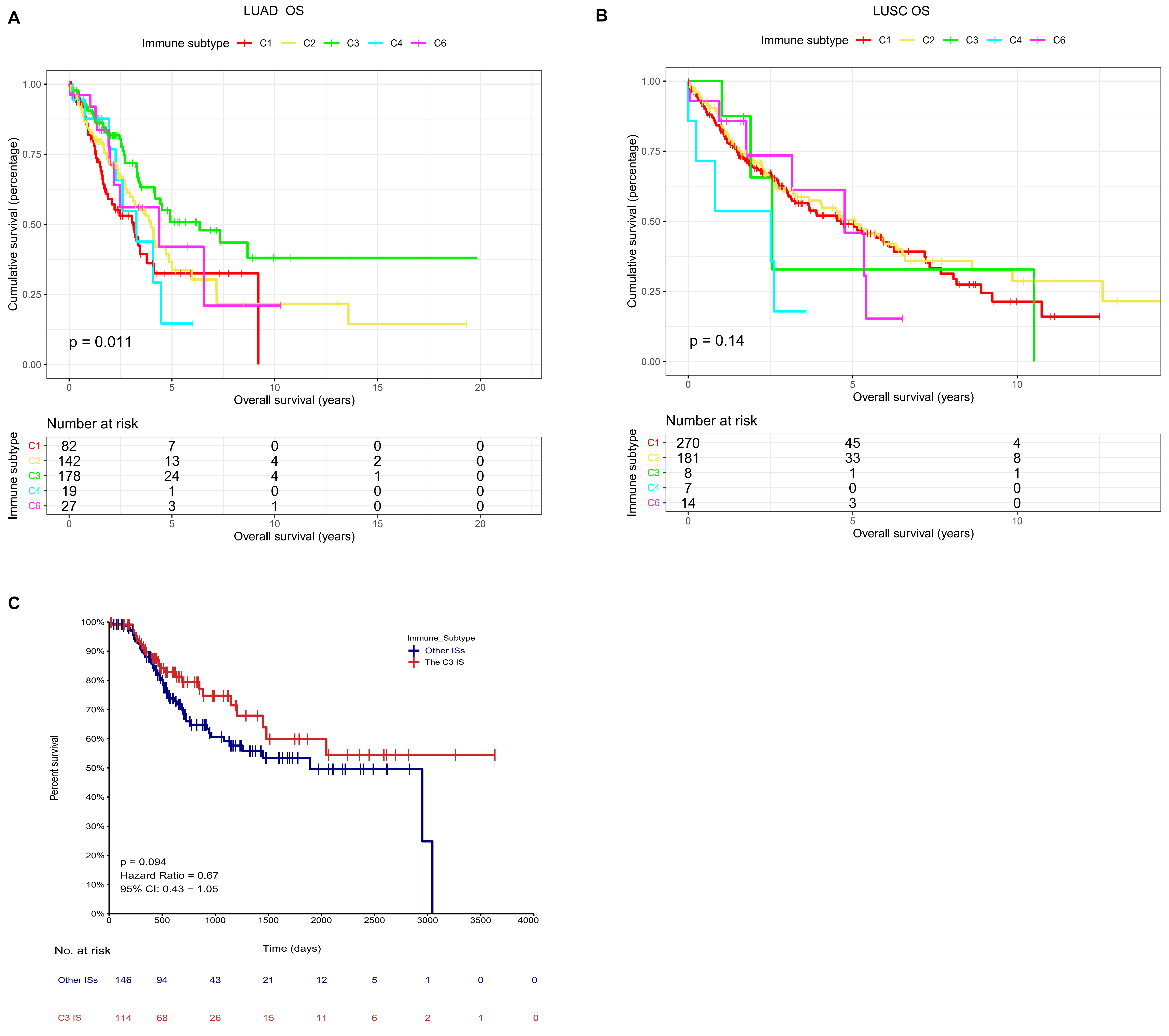
A
LUAD OS
Immune subtype C1 C2 C3 C4 C6
Cumulative survival (percentage)
1.00
0.75
0.50
0.25
0.00
p = 0.011
0 5 10 15 20
Overall survival (years)
Number at risk
Immune subtype
C1 82 7 0 0 0
C2 142 13 4 2 0
C3 178 24 4 1 0
C4 19 1 0 0 0
C6 27 3 1 0 0
0 5 10 15 20
Overall survival (years)
B
LUSC OS
Immune subtype C1 C2 C3 C4 C6
Cumulative survival (percentage)
1.00
0.75
0.50
0.25
0.00
p = 0.14
0 5 10
Overall survival (years)
Number at risk
Immune subtype
C1 270 45 4
C2 181 33 8
C3 8 1 1
C4 7 0 0
C6 14 3 0
0 5 10
Overall survival (years)
C
Immune_Subtype
Other ISs
The C3 IS
Percent survival
100%
90%
80%
70%
60%
50%
40%
30%
20%
10%
0%
p = 0.094
Hazard Ratio = 0.67
95% CI: 0.43 − 1.05
0 500 1000 1500 2000 2500 3000 3500 4000
Time (days)
No. at risk
Other ISs 146 94 43 21 12 5 1 0 0
C3 IS 114 68 26 15 11 6 2 1 0

Figure S2

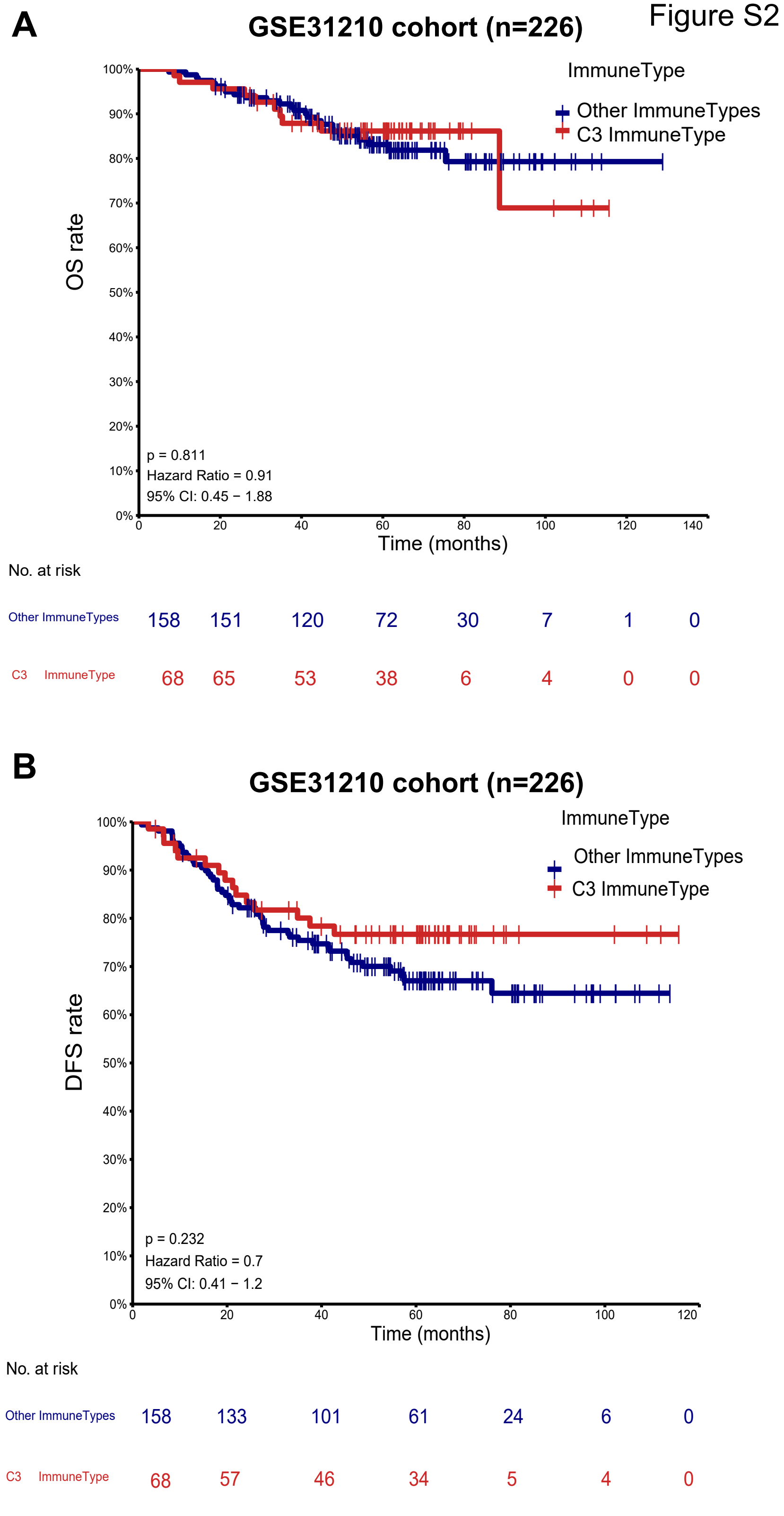
A
GSE31210 cohort (n=226)
ImmuneType
Other ImmuneTypes
C3 ImmuneType
OS rate
100%
90%
80%
70%
60%
50%
40%
30%
20%
10%
0%
p = 0.811
Hazard Ratio = 0.91
95% CI: 0.45 − 1.88
0
20
40
60
80
100
120
140
Time (months)
No. at risk
Other ImmuneTypes 158 151 120 72 30 7 1 0
C3 ImmuneType 68 65 53 38 6 4 0 0
B
GSE31210 cohort (n=226)
ImmuneType
Other ImmuneTypes
C3 ImmuneType
DFS rate
100%
90%
80%
70%
60%
50%
40%
30%
20%
10%
0%
p = 0.232
Hazard Ratio = 0.7
95% CI: 0.41 − 1.2
0
20
40
60
80
100
120
Time (months)
No. at risk
Other ImmuneTypes 158 133 101 61 24 6 0
C3 ImmuneType 68 57 46 34 5 4 0

Figure S3

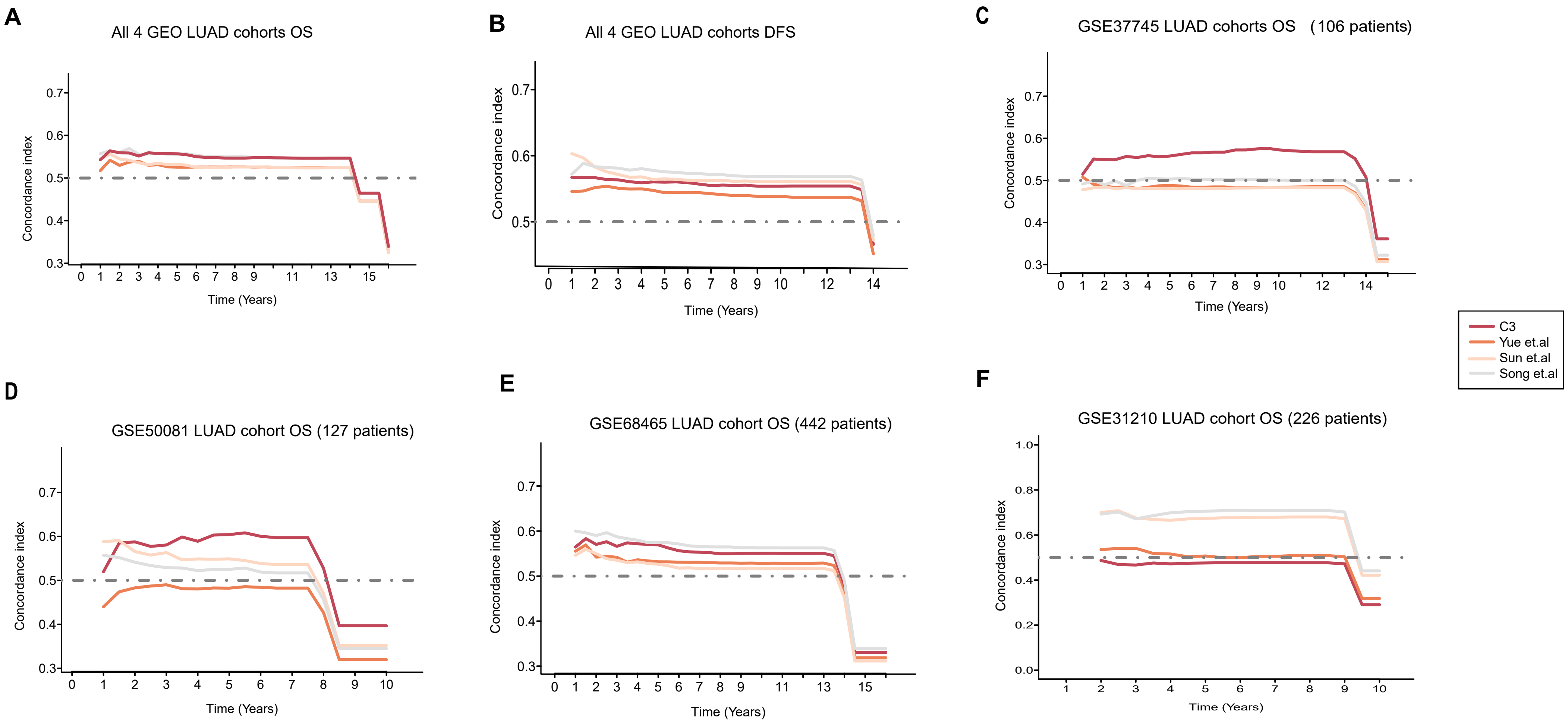
A
All 4 GEO LUAD cohorts OS
Concordance index
0.7
0.6
0.5
0.4
0.3
0 1 2 3 4 5 6 7 8 9 11 13 15
Time (Years)
B
All 4 GEO LUAD cohorts DFS
Concordance index
0.7
0.6
0.5
0 1 2 3 4 5 6 7 8 9 10 12 14
Time (Years)
C
GSE37745 LUAD cohorts OS (106 patients)
Concordance index
0.7
0.6
0.5
0.4
0.3
0 1 2 3 4 5 6 7 8 9 10 12 14
Time (Years)
C3
Yue et.al
Sun et.al
Song et.al
D
GSE50081 LUAD cohort OS (127 patients)
Concordance index
0.7
0.6
0.5
0.4
0.3
0 1 2 3 4 5 6 7 8 9 10
Time (Years)
E
GSE68465 LUAD cohort OS (442 patients)
Concordance index
0.7
0.6
0.5
0.4
0.3
0 1 2 3 4 5 6 7 8 9 11 13 15
Time (Years)
F
GSE31210 LUAD cohort OS (226 patients)
Concordance index
1.0
0.8
0.6
0.4
0.2
0.0
1 2 3 4 5 6 7 8 9 10
Time (Years)

Figure S4

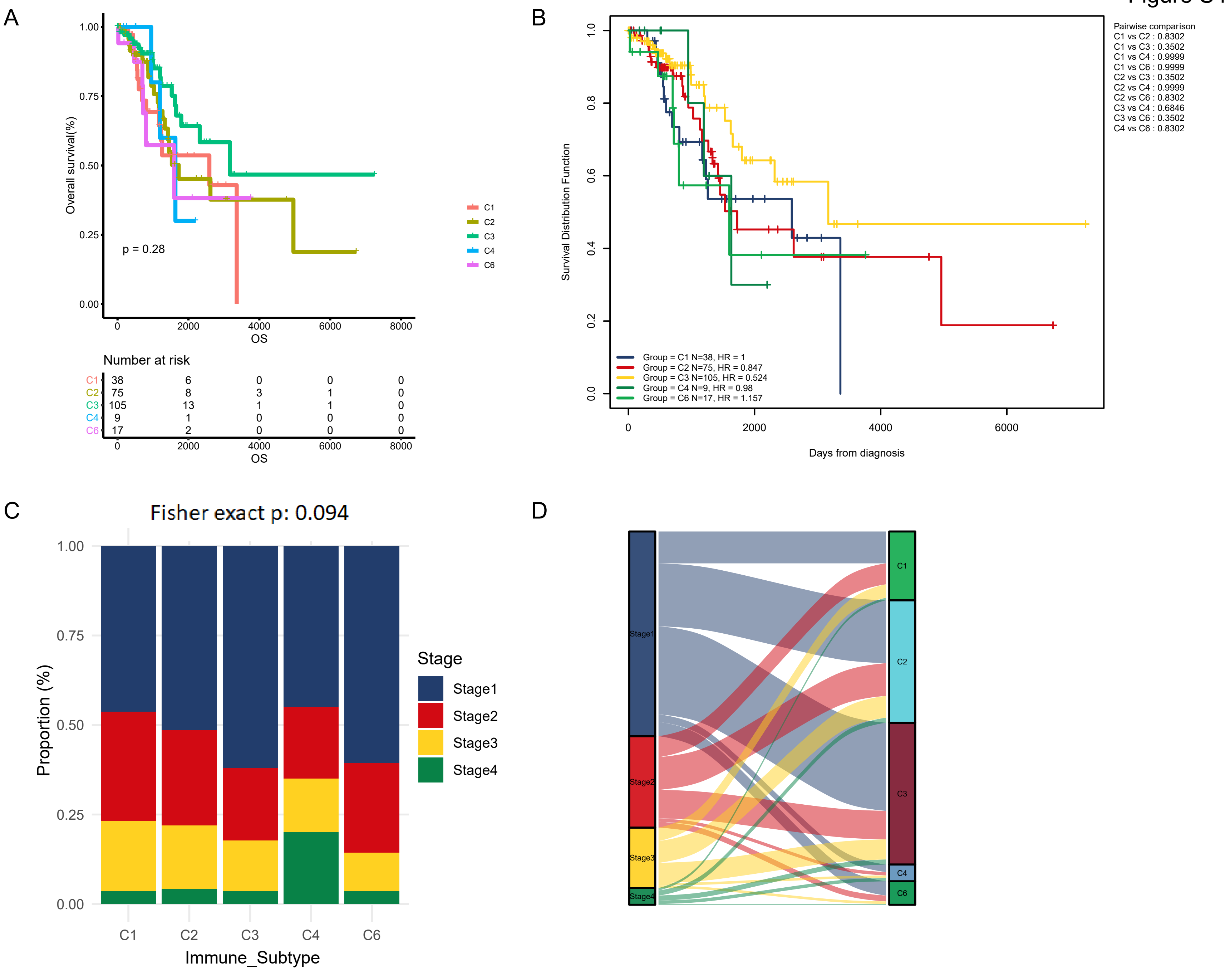
A
1.00
0.75
0.50
0.25
0.00
Overall survival(%)
p = 0.28
0
2000
4000
6000
8000
OS
C1
C2
C3
C4
C6
Number at risk
C1 38 6 0 0 0
C2 75 8 3 1 0
C3 105 13 1 1 0
C4 9 1 0 0 0
C6 17 2 0 0 0
B
Survival Distribution Function
Days from diagnosis
Pairwise comparison
C1 vs C2 : 0.8302
C1 vs C3 : 0.3502
C1 vs C4 : 0.9999
C1 vs C6 : 0.9999
C2 vs C3 : 0.3502
C2 vs C4 : 0.9999
C2 vs C6 : 0.8302
C3 vs C4 : 0.6846
C3 vs C6 : 0.3502
C4 vs C6 : 0.8302
Group = C1 N=38, HR = 1
Group = C2 N=75, HR = 0.847
Group = C3 N=105, HR = 0.524
Group = C4 N=9, HR = 0.98
Group = C6 N=17, HR = 1.157
C
Fisher exact p: 0.094
Proportion (%)
Immune_Subtype
Stage
Stage1
Stage2
Stage3
Stage4
D
Stage1
Stage2
Stage3
Stage4
C1
C2
C3
C4
C6

Figure S2

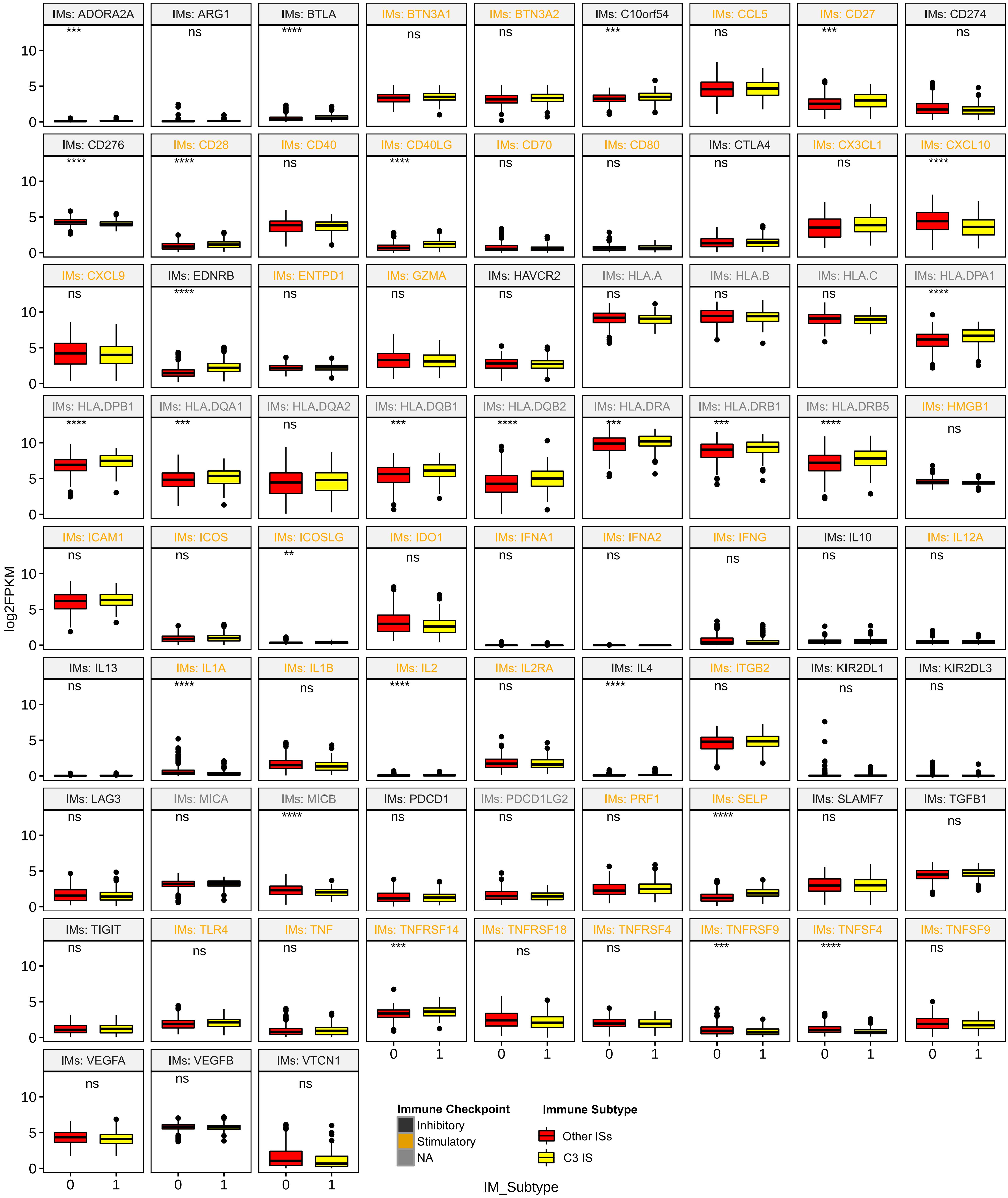

IMs: ADORA2A
IMs: ARG1
IMs: BTLA
IMs: BTN3A1
IMs: BTN3A2
IMs: C10orf54
IMs: CCL5
IMs: CD27
IMs: CD274
IMs: CD276
IMs: CD28
IMs: CD40
IMs: CD40LG
IMs: CD70
IMs: CD80
IMs: CTLA4
IMs: CX3CL1
IMs: CXCL10
IMs: CXCL9
IMs: EDNRB
IMs: ENTPD1
IMs: GZMA
IMs: HAVCR2
IMs: HLA.A
IMs: HLA.B
IMs: HLA.C
IMs: HLA.DPA1
IMs: HLA.DPB1
IMs: HLA.DQA1
IMs: HLA.DQA2
IMs: HLA.DQB1
IMs: HLA.DQB2
IMs: HLA.DRA
IMs: HLA.DRB1
IMs: HLA.DRB5
IMs: HMGB1
IMs: ICAM1
IMs: ICOS
IMs: ICOSLG
IMs: IDO1
IMs: IFNA1
IMs: IFNA2
IMs: IFNG
IMs: IL10
IMs: IL12A
IMs: IL13
IMs: IL1A
IMs: IL1B
IMs: IL2
IMs: IL2RA
IMs: IL4
IMs: ITGB2
IMs: KIR2DL1
IMs: KIR2DL3
IMs: LAG3
IMs: MICA
IMs: MICB
IMs: PDCD1
IMs: PDCD1LG2
IMs: PRF1
IMs: SELP
IMs: SLAMF7
IMs: TGFB1
IMs: TIGIT
IMs: TLR4
IMs: TNF
IMs: TNFRSF14
IMs: TNFRSF18
IMs: TNFRSF4
IMs: TNFRSF9
IMs: TNFSF4
IMs: TNFSF9
IMs: VEGFA
IMs: VEGFB
IMs: VTCN1
log2FPKM
IM_Subtype
Immune Checkpoint
Inhibitory
Stimulatory
NA
Immune Subtype
Other ISs
C3 IS

Figure S6

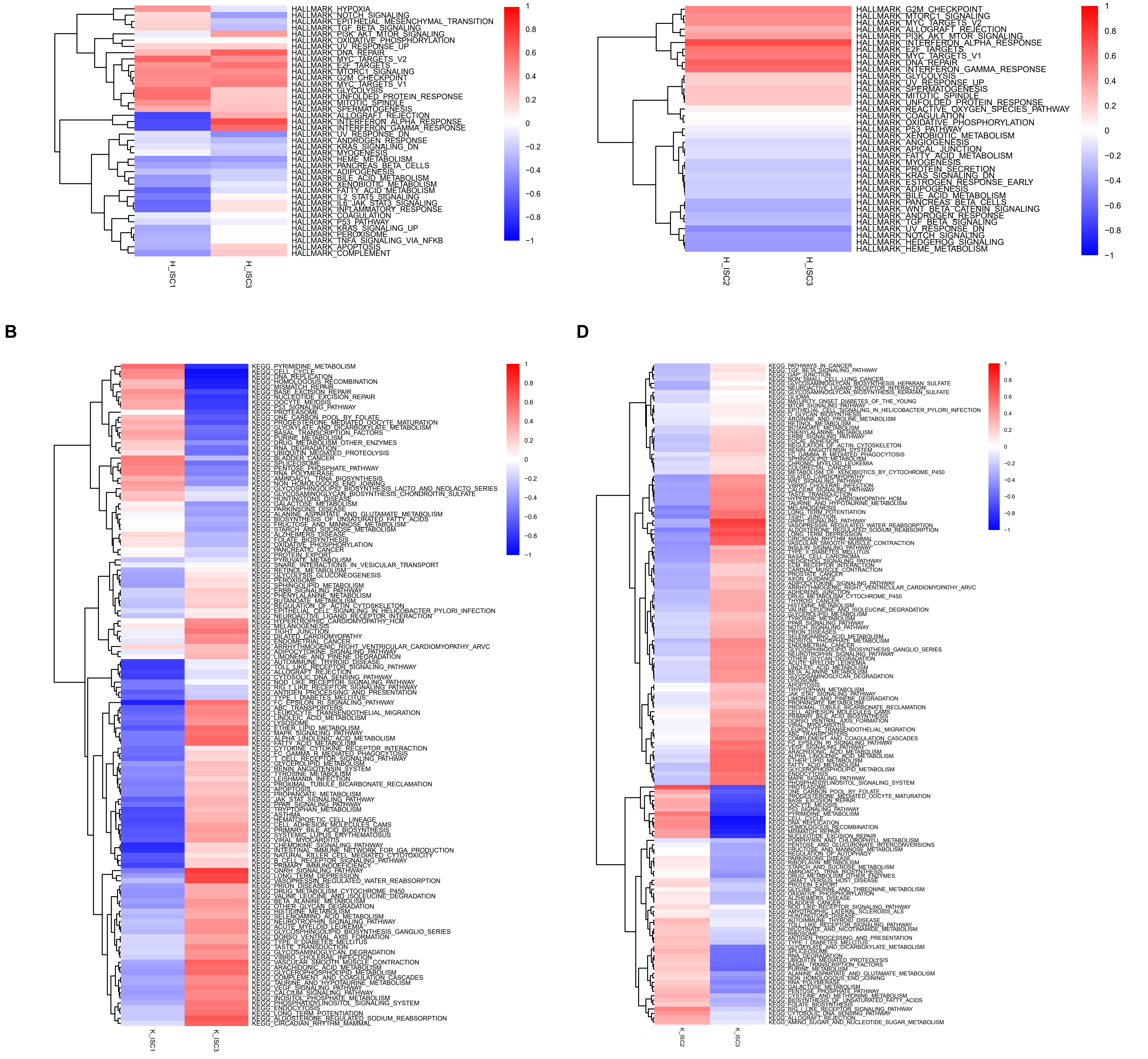
A
HALLMARK_HYPOXIA
HALLMARK_NOTCH_SIGNALING
HALLMARK_EPITHELIAL_MESENCHYMAL_TRANSITION
HALLMARK_TGF_BETA_SIGNALING
HALLMARK_PI3K_AKT_MTOR_SIGNALING
HALLMARK_OXIDATIVE_PHOSPHORYLATION
HALLMARK_UV_RESPONSE_UP
HALLMARK_DNA_REPAIR
HALLMARK_MYC_TARGETS_V2
HALLMARK_E2F_TARGETS
HALLMARK_MTORC1_SIGNALING
HALLMARK_G2M_CHECKPOINT
HALLMARK_MYC_TARGETS_V1
HALLMARK_GLYCOLYSIS
HALLMARK_UNFOLDED_PROTEIN_RESPONSE
HALLMARK_MITOTIC_SPINDLE
HALLMARK_SPERMATOGENESIS
HALLMARK_ALLOGRAFT_REJECTION
HALLMARK_INTERFERON_ALPHA_RESPONSE
HALLMARK_INTERFERON_GAMMA_RESPONSE
HALLMARK_UV_RESPONSE_DN
HALLMARK_ANDROGEN_RESPONSE
HALLMARK_KRAS_SIGNALING_DN
HALLMARK_MYOGENESIS
HALLMARK_HEME_METABOLISM
HALLMARK_PANCREAS_BETA_CELLS
HALLMARK_ADIPOGENESIS
HALLMARK_BILE_ACID_METABOLISM
HALLMARK_XENOBIOTIC_METABOLISM
HALLMARK_FATTY_ACID_METABOLISM
HALLMARK_IL2_STAT5_SIGNALING
HALLMARK_IL6_JAK_STAT3_SIGNALING
HALLMARK_INFLAMMATORY_RESPONSE
HALLMARK_COAGULATION
HALLMARK_P53_PATHWAY
HALLMARK_KRAS_SIGNALING_UP
HALLMARK_PEROXISOME
HALLMARK_TNFA_SIGNALING_VIA_NFKB
HALLMARK_APOPTOSIS
HALLMARK_COMPLEMENT
H_ISC1
H_ISC3
1
0.8
0.6
0.4
0.2
0
−0.2
−0.4
−0.6
−0.8
−1
C
HALLMARK_G2M_CHECKPOINT
HALLMARK_MTORC1_SIGNALING
HALLMARK_MYC_TARGETS_V2
HALLMARK_ALLOGRAFT_REJECTION
HALLMARK_PI3K_AKT_MTOR_SIGNALING
HALLMARK_INTERFERON_ALPHA_RESPONSE
HALLMARK_E2F_TARGETS
HALLMARK_MYC_TARGETS_V1
HALLMARK_DNA_REPAIR
HALLMARK_INTERFERON_GAMMA_RESPONSE
HALLMARK_GLYCOLYSIS
HALLMARK_UV_RESPONSE_UP
HALLMARK_SPERMATOGENESIS
HALLMARK_MITOTIC_SPINDLE
HALLMARK_UNFOLDED_PROTEIN_RESPONSE
HALLMARK_REACTIVE_OXYGEN_SPECIES_PATHWAY
HALLMARK_COAGULATION
HALLMARK_OXIDATIVE_PHOSPHORYLATION
HALLMARK_P53_PATHWAY
HALLMARK_XENOBIOTIC_METABOLISM
HALLMARK_ANGIOGENESIS
HALLMARK_APICAL_JUNCTION
HALLMARK_FATTY_ACID_METABOLISM
HALLMARK_MYOGENESIS
HALLMARK_PROTEIN_SECRETION
HALLMARK_KRAS_SIGNALING_DN
HALLMARK_ESTROGEN_RESPONSE_EARLY
HALLMARK_ADIPOGENESIS
HALLMARK_BILE_ACID_METABOLISM
HALLMARK_PANCREAS_BETA_CELLS
HALLMARK_WNT_BETA_CATENIN_SIGNALING
HALLMARK_ANDROGEN_RESPONSE
HALLMARK_TGF_BETA_SIGNALING
HALLMARK_UV_RESPONSE_DN
HALLMARK_NOTCH_SIGNALING
HALLMARK_HEDGEHOG_SIGNALING
HALLMARK_HEME_METABOLISM
H_ISC2
H_ISC3
1
0.8
0.6
0.4
0.2
0
−0.2
−0.4
−0.6
−0.8
−1
B
K_ISC1
K_ISC3
D
K_ISC2
K_ISC3

Figure S7

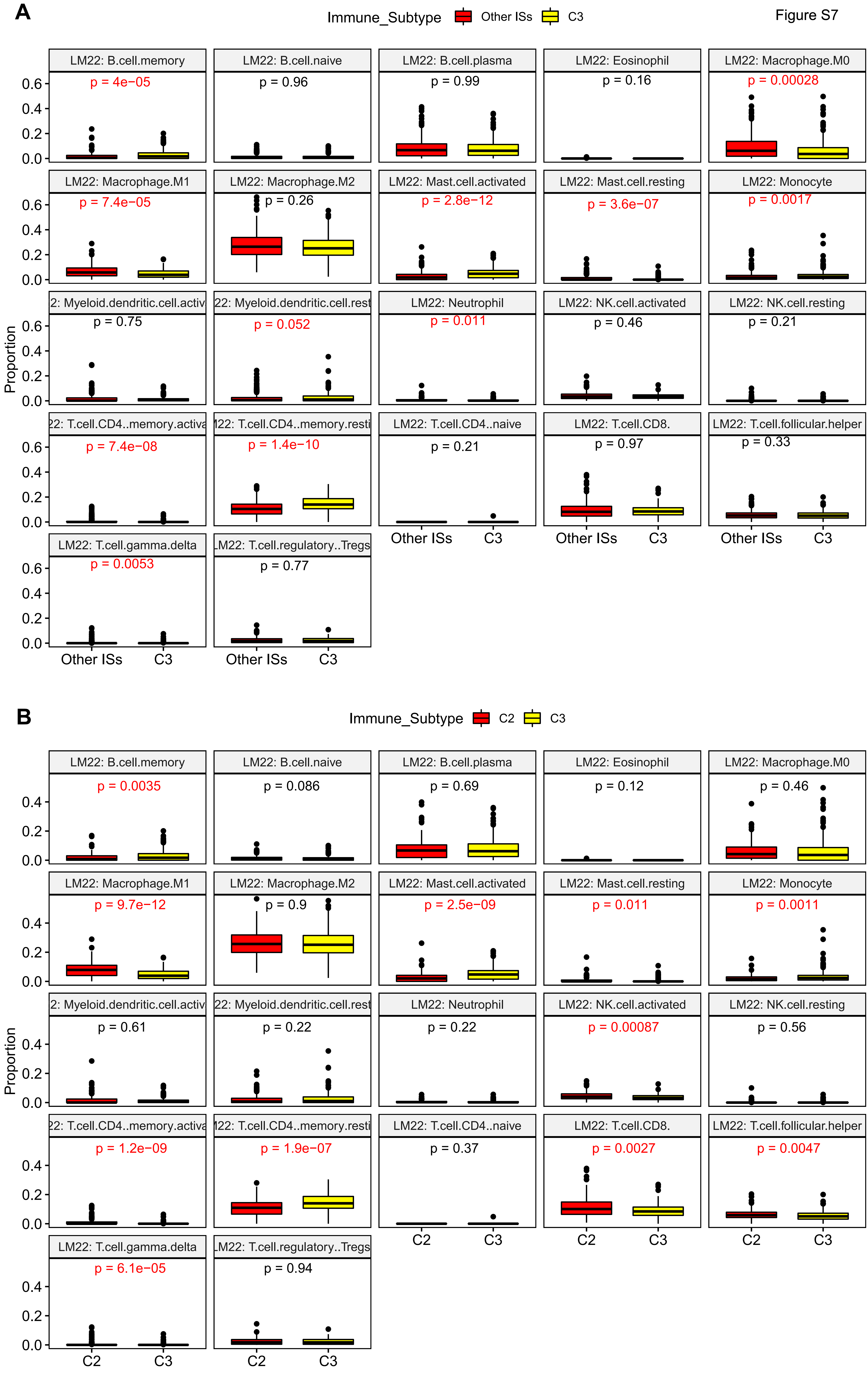
A
Immune_Subtype
Other ISs
C3
Proportion
LM22: B.cell.memory
p = 4e−05
LM22: B.cell.naive
p = 0.96
LM22: B.cell.plasma
p = 0.99
LM22: Eosinophil
p = 0.16
LM22: Macrophage.M0
p = 0.00028
LM22: Macrophage.M1
p = 7.4e−05
LM22: Macrophage.M2
p = 0.26
LM22: Mast.cell.activated
p = 2.8e−12
LM22: Mast.cell.resting
p = 3.6e−07
LM22: Monocyte
p = 0.0017
2: Myeloid.dendritic.cell.activ
p = 0.75
22: Myeloid.dendritic.cell.rest
p = 0.052
LM22: Neutrophil
p = 0.011
LM22: NK.cell.activated
p = 0.46
LM22: NK.cell.resting
p = 0.21
22: T.cell.CD4..memory.activa
p = 7.4e−08
122: T.cell.CD4..memory.resti
p = 1.4e−10
LM22: T.cell.CD4..naive
p = 0.21
LM22: T.cell.CD8.
p = 0.97
LM22: T.cell.follicular.helper
p = 0.33
LM22: T.cell.gamma.delta
p = 0.0053
LM22: T.cell.regulatory..Tregs
p = 0.77
B
Immune_Subtype
C2
C3
Proportion
LM22: B.cell.memory
p = 0.0035
LM22: B.cell.naive
p = 0.086
LM22: B.cell.plasma
p = 0.69
LM22: Eosinophil
p = 0.12
LM22: Macrophage.M0
p = 0.46
LM22: Macrophage.M1
p = 9.7e−12
LM22: Macrophage.M2
p = 0.9
LM22: Mast.cell.activated
p = 2.5e−09
LM22: Mast.cell.resting
p = 0.011
LM22: Monocyte
p = 0.0011
2: Myeloid.dendritic.cell.activ
p = 0.61
22: Myeloid.dendritic.cell.rest
p = 0.22
LM22: Neutrophil
p = 0.22
LM22: NK.cell.activated
p = 0.00087
LM22: NK.cell.resting
p = 0.56
22: T.cell.CD4..memory.activa
p = 1.2e−09
122: T.cell.CD4..memory.resti
p = 1.9e−07
LM22: T.cell.CD4..naive
p = 0.37
LM22: T.cell.CD8.
p = 0.0027
LM22: T.cell.follicular.helper
p = 0.0047
LM22: T.cell.gamma.delta
p = 6.1e−05
LM22: T.cell.regulatory..Tregs
p = 0.94
